# Low prevalence of active trachoma and associated factors among children aged 1–9 years in rural communities of Metema District, Northwest Ethiopia: a community based cross-sectional study

**DOI:** 10.1186/s13052-021-01064-x

**Published:** 2021-05-17

**Authors:** Kessete Ayelgn, Tadesse Guadu, Atalay Getachew

**Affiliations:** 1 Amhara Region West Gondar Health Department, Northwest Ethiopia, Gondar, Ethiopia; 2 Department of Environmental and Occupational Health and Safety, Institute of Public Health, College of Medicine and Health Sciences, University of Gondar, Gondar, Ethiopia; 3 Department of Environmental Health, College of Medicine and Health Sciences, Debre Markos University, Debre Markos, Ethiopia

**Keywords:** Prevalence, Active trachoma, Children, Metema district

## Abstract

**Background:**

Trachoma is an infectious disease of the eye caused by *Chlamydia trachomatis* and transmitted via contact with eye discharge from infected persons and leading to blindness worldwide. Children less than 9 years of age affected more seriously. The disease is common where access to water and sanitation are limited.

**Objective:**

To determine the prevalence of active trachoma and associated factors among children aged 1–9 years in rural communities of Metema District, West Gondar Zone, Northwest Ethiopia.

**Method:**

A community based cross-sectional study design was used to collect data from 792 children aged 1–9 years old in Metema district from April to May 2018. Multistage sampling technique was used to select the study participants. Pretested interviewer-administered structured questionnaire and eye examination using binocular loupe to differentiate trachoma cases was the data collection methods and tools. The bivariable and multivariable binary logistic regression model was employed for analysis. *P*-value < 0.05 was considered to declare statistical significance.

**Results:**

A total of 752 children aged l-9 years were enrolled in this study with response rate of 94.9%. The overall prevalence of active trachoma among the study participants was 11.8% (95% CI, 9.5–13.9). Unprotected source of water (AOR = 4.7; 95% CI: 2.5–8.9), lower household water consumption (AOR = 2.8; 95% CI: 1.3–6.0), improper latrine utilization (AOR = 3.2; 95% CI: 1.5–6.7), and frequency of face washing once per day (AOR = 5.3; 95% CI: 1.2–26.6) were the factors significantly associated with active trachoma.

**Conclusion:**

The current study revealed a lower overall prevalence of active trachoma (11.8%) than the WHO threshold prevalence (20%) used to declare it as a severe public health problem. All residents and health professional should collaborate on trachoma prevention by implementing the WHO SAFE strategy- surgery for trichiasis, antibiotics, facial cleanliness and environmental improvement for further trachoma elimination.

## Background

Trachoma is an infectious disease of the eye caused by *Chlamydia trachomatis* and transmitted via contact with eye discharge from infected persons and leading to blindness worldwide [1]. Trachoma contributes for significant public health problem in many part of the world including Europe and North America [2]. According to World Health Organization (WHO) report estimated in 55 countries, trachoma is endemic [3, 4]. Active trachoma is common in area where trachoma is endemic. High prevalence occurs mainly in Africa, Asia, Middle East, Latin America and Australia [5]. It is also estimated that 84 million people have active trachoma [2].

About 49% of the global burden of active trachoma is highly distributed in 5 countries, where Ethiopia was one of them [2, 6]. In Ethiopia one nationwide survey on blindness, low vision, and trachoma reported that the overall national prevalence of active trachoma among children 1 up to 9 years old was 40.14%.The highest prevalence was observed (62.6%) in Amhara region [7].

The transmission of trachoma has been known to occur by direct contact with eye-seeking fly Musca sorbens which lays its eggs on exposed human feces [8]. Infection with trachoma is most commonly found in children and with repeated re-infection it can lead to scarring complications and blindness in late childhood and adult life [9–12]. The WHO simplified grading scheme comprises five signs. For programmers planning, monitoring and evaluation, three of these five signs are particularly important: Trachomatous inflammation follicles (TF), Trachomatous inflammation trichiasis (TT), and corneal opacity (CO). The prevalence of TF in children aged 1–9 years is the key index for determining whether an area needs intervention with the A (Antibiotics to clear infection), F (Facial cleanliness) and E (Environmental improvement) components of SAFE based on WHO recommendation. The prevalence of TT determines the probable need for surgical services. The prevalence of CO is a (rough) measure of the burden of blindness and visual impairment due to trachoma [2, 13].

The risk factors for trachoma vary between place to place depending on economic, behavioral and environmental factors. Unclean faces, or clean faces but with flies, time to fetch water, overcrowding, garbage within the compound, less frequent face washing, practice of open defecation, household cattle ownership, high household fly density and long distance to the nearest water source are among the factors that have been associated with active trachoma [7].

Children less than 9 years are the major reservoir of the bacteria while children under this age cannot take care of themselves and characterized by unclean faces, foods on faces, dust, and nasal discharge that attract eye-seeking flies those carriers bacteria [14]. Furthermore, children under 9 years are more likely to touch their eyes more often thus enhance auto-reinjection of Trachoma. The problem of trachoma is common where access to water and sanitation is limited [3]. Lack of such kind of facility can occur in rural than urban community. The majority, 90% community of Metema district lives in rural area. There is a scarcity of information on the prevalence of active trachoma in Metema district. Therefore, the aim of this study was to determine the prevalence of active trachoma and associated factors among children aged 1–9 years in rural communities of Metema district, Northwest Ethiopia.

## Methods and materials

### Study design and period

A community based cross-sectional study design was employed to determine the prevalence of active trachoma and associated factors among children aged 1–9 years in rural communities of Metema district, from April to May 2018.

### Study area

This study was conducted in Metema district, West Gondar Zone, Northwest Ethiopia. Metema Districtis located 170 km from Gondar town, in the border of South Sudan. There are 17 rural and 2 urban *kebeles* in the district. Moreover, it has 5 Health center and 19 heath post. Total projected populations of the district are 149,700. Of this total population, Children aged 1–9 years are 26,373. The major source of income of population is agricultural activities especially production sesame and it is known for investment district in the country.

### Source and study population

All children in Metema district whose ages between 1 and 9 years were the source population. Children who are living in 6 kebeles selected by simple random sampling technique were the study population, while children who were living in households selected by systematic random sampling technique were the study subjects. Children who were unable to undergo physical examination for trachoma evaluation due to serious sickness, and those absent during the data collection period were excluded from the study.

### Study variables

#### Dependent variable

Active trachoma (Yes/No).

#### Independent variables

##### Socio-demographic factors

family size, education level, occupation, Income, Age of household, Sex of household,Religion, Sex of child and Age of child,number of animal.

##### Environmental factors

Source of water, water consumption, distance to water source, latrien availablity, latrien utilization, waste disposal practice, animal keeping practice, and cleanliness of compounds.

##### Behavioral factors

Frequency**:** Face washing**,** frequency of washing, Use of soap, eye discharge, Nasal discharge, fly on face, Number of fly’s on children face, facial cleanness.

#### Sample size determination and sampling procedures

The sample size for the prevalence of active trachoma was determined using single population proportion formula with the assumptions; proportion (P) of trachoma 62.6% [15], 95% confidence interval (CI), margin of error of 5%, design effect 2 and adding 10% contingency making a total sample size of 792.

For associated factors of prevalence of active trachoma, the sample size was computed using double population proportion formula using EpiInfo ver.7 software by considering the different variables like distance to water source, use of soap, number of fly’s on children face and facial cleanness. But, the sample sizes were low compared to the sample size for the prevalence of active trachoma. Finally, the largest sample size was taken for this study.

A multistage sampling technique was applied to select study subject. At first stage, 6 out of 17 rural *kebeles* were selected by lottery method. Using probability proportional to size (PPS), the number of households was determined in each kebeles. At the second stage, systematic random sampling technique was applied to select study households. One child per household was included in the study. In case, where there are more than one child with the age of 1–9 years in the same household, lottery method was used to select a child.

#### Operational definitions

##### Presence of active trachoma

Is the presence of Trachomatous inflammation, follicles (the presence of at least five or more follicles at least 0.5 mm in Diameter in the central part of the upper tarsal conjunctiva) or Trachomatous inflammation intense (pronounced inflammatory thickening of the tarsal conjunctiva that obscures more than half of the normal deep tarsal vessels) [16].

##### Clean face

A child who did not have an eye discharge or nasal discharge, fly on face at the time of visit.

##### Number of flies

Presence of flies on children’s faces for about 3 s during the examination time which was graded as none (0 flies), few (1–4 flies), or many (≥5 flies).

#### Data collection tools and procedure

A structured and pre-tested interviewer-administered questionnaire, and eye examination by using binocular loupe to differentiate trachoma cases using observation checklist were used to collect data. The questionnaire was first prepared in English and translated to Amharic, and then again translated back to English by another person. The questionnaire has information on socio-demographic characteristics, Environmental factors and behavioral factors. A total of 10 health professionals were participated in the study among this 2 supervisors were assigned to the data collection for head of household interviews and validate trachoma grading. Trachoma grading was be examine by 2 trend health professional, according to the WHO simplified grading scheme [17]. The rest 6 health professionals were interview head of the households.

Before the commencement of data collection, 3 days training was given for all data collectors, trachoma examiners and supervisors. Moreover, before the actual data collection, the examination of eye with questionnaire was pre-tested on 5% (40) of final sample size in an adjacent *kebele* outside of the study area. During the course of the data collection, data collectors were intensively supervised at each site. The completeness and accuracy of data was checked at the end of each day.

#### Data management and analysis

The data was entered using Epi Info ver.7 software by the principal investigator and exported to SPSS version 20 for analysis. Descriptive statistics were used to describe the socio-demographic characteristics of the respondents, the prevalence of trachoma, and other characteristics of the respondents. The bivariable and multivariable binary logistic regression model was used to assess the association between dependent and independent variables. Hosmer-Lemeshow goodness of fit test was used to check model fitness (*P* > =0.05)**.** Variables having *P*-value ≤0.2 in the bivariable analysis were further entered into the multivariable analysis to control the effects of confounders. In the multivariable analysis, *P*-value < 0.05 was considered to declare statistical significance.

#### Ethical considerations

Ethical clearance was obtained from the Institutional Review Board of the University of Gondar, College of Medicine and Health Science, Institute of public health. Support letter and permission letter were obtained from Metema District Health Office. The study participants were provided full information regarding the purpose and nature of the research then written consent was obtained from each parent or caregiver of participants. Participation in the study was on a voluntary basis, and participants were informed about their right not to participate in the study or withdraw at any time. Moreover, the confidentiality of the information was assured by using an anonymous questionnaire. All identified cases of active trachoma were provided with the standard treatment free of charge and complicated cases were referred to the nearest health center for better treatment.

## Results

### Socio-demographic characteristics

A total of 752 children aged 1–9 years participated in the study, giving response rate of 94.9%. The mean ages of the sampled children were 1.9 [SD ± 0.8] years. Majority of the study subjects 72.6% (546), live in households headed by male and 37.2% (380) of heads of households were farmers by occupation. More than half, 55.6% (418) of the head of households were illiterate, 60.6% (456) had a family size of more than five, and 57.1% (353) of the children were females. The age distribution showed that 38.0% (286) were within the age group between 1 and 3 years (Table 1).

**Table 1:** Socio demographic characteristics of the study participants in Metema District, Northwest Ethiopia, 2018 (*n* = 752)

Variable	Frequency (N)	Percentage (%)
**Head of household**		
Male	546	72.6
Female	206	27.4
**Age of head of household**		
18–29	142	19.0
30–44	518	68.9
45–59	91	12.1
**Occupation**		
Farmer	380	37.2
Daily laborer	230	30.6
Merchant	242	32.2
**Level of Education**		
Illiterate	418	55.6
Primary and above	334	44.4
**Average monthly income**		
less than 600	132	17.6
601–1200	230	30.6
1201–2000	207	27.5
above 2000	183	24.3
**Religion**		
Orthodox	394	52.4
Muslim	358	47.6
**Family size**		
≤ 5	296	39.4
> 5	456	60.6
**Sex of child**		
Male	352	46.8
Female	400	53.2
**Age of children’s**		
1–3	286	38.0
4–6	250	33.2
7–9	216	28.7

### Environmental characteristics of the households

Majority of the households, 73.5% (553) obtain their water from protected source and 66.4% (499) took less than 30 min to collect water. About 40.4% (304) of the families’ average water consumption was 60–80 l per head. The majority of the households, 90.4% (680) had latrine. From these 62.1% (467) of households, practice proper utilization of latrine. More than half of the households, 57.6% (433) practice proper disposal of garbage and compound cleanses (Table 2).

**Table 2:** Environmental characteristics of selected households in Metema District, Northwest Ethiopia, 2018 (*n* = 752)

Variable	Number (N)	Percent (%)
**Source of Water**		
Protected source	553	73.5
Unprotected source	199	26.5
**Distance to collect water (round trip)**		
< =30 min	499	66.4
> 30 min	253	33.6
**Average water consumption**		
40–60 l per head	199	26.5
60–80 l per head	304	40.4
Above 80 l per head	249	33.1
**Accesses of latrine**		
No	72	9.6
Yes	680	90.4
**Latrine utilization**		
Improper utilization	285	37.9
Proper utilization	467	62.1
**Disposal of garbage**		
Improper disposal	319	42.4
Proper disposal	433	57.6
**Compound cleanses**		
No	268	35.6
Yes	484	64.6
**Animal keeping practice separately at home**		
Kept only at night	18	2.4
Kept both night and day	734	97.6

### Behavioral characteristics

Majority of children’s, 79.3% (596) wash their face once per day and 83.5% (628) of them use soap for washing. Only 5.5% (41) and 8.9% (67) of the children had eye discharge and nasal discharge, respectively. The majority of the children, 83.5% (*N* = 628) had clean face (Table 3).

**Table 3:** Face washing habit and Facial cleanliness characteristics of children’s in Metema District, Northwest Ethiopia, 2018 (*n* = 752)

Variable	Frequency(N)	Percent (%)
**Frequency of washing**		
One per day	596	79.3
Twice per day	156	20.7
**Use of soap**		
No	124	16.5
Yes	628	83.5
**Eye discharge**		
No	711	94.5
Yes	41	5.5
**Nasal discharge**		
No	685	91.1
Yes	67	8.9
**Fly on child face**		
No	643	85.5
Yes	109	14.5
**Number of fly**		
0	643	85.5
1–4	109	14.5
**Facial cleanness**		
Clean	628	83.5
Unclean	124	16.5

### Prevalence of active trachoma

The overall prevalence of active trachoma among children aged 1–9 years in rural communities of Metema district was 11.8% (95% CI, 9.5–14.1). Amongst the examined children, the prevalence of TF and TI was 9.4 and 2.4%, respectively. Furthermore, the prevalence difference among sex and age group was 5.8 and 6% for male and female and 4.6, 3.3 and 3.9% for children 1–3, 4–6 and 7–9 age groups, respectively.

### Factors associated with active trachoma

The multivariate logistic regression analysis showed that unprotected source of water (AOR = 4.7; 95% CI: 2.5–8.9), lower household water consumption (AOR =2.8; 95% CI: 1.3–6.0), improper latrine utilization (AOR = 3.3; 95% CI: 1.5–6.7), and frequency of face washing once per day (AOR = 5.3; 95% CI: 1.2–26.6) were the factors associated with active trachoma (Table 4).

**Table 4:** Bivariate and multivariate analysis of factors associated with active trachoma among children’s in Metema District, Northwest Ethiopia 2018 (*n* = 752)

Variable	Active Trachoma Yes (%) No (%)		COR (95% CI)	AOR (95% CI)
**Family size**				
> 5	71	385	2.8 (1.7–4.9)	1.74 (0.8–4.0)
≤ 5	18	278	1.00	1.00
**Education status**				
Illiterates	73	345	4.2 (2.4–7.4)	2.3 (1.0–5.4)
Primary & above	16	318	1.00	1.00
**Source of water**				
unprotected source	65	134	10.7 (6.5–17.7)	4.7 (2.5–8.9)***
Protected source	24	529	1.00	1.00
**Distance to water source**				
> 30 min	63	190	6.0 (3.7–9.8)	1.8 (0.9–3.3)
≤ 30 min	26	473	1.00	1.00
**Water consumption**				
40–60 l per HH	52	147	5.9 (3.2–11.1)	2.8 (1.3–6.0)**
60–80 l per HH	23	281	1.4 (0.7–2.7)	0.8 (0.4–1.9)
> 80 l per HH	14	235	1.00	1.00
**Disposal of garbage**.				
Improper	67	252	5.0 (3.0–8.2)	1.5 (0.8–2.9)
Proper	22	411	1.00	1.00
**Latrine utilization**				
Improper	76	209	12.7 (6.9–23.4)	3.2 (1.5–6.7)***
Proper	13	454	1.00	1.00
**Frequency of face washing**				
one per day	87	509	13.2 (3.2–54.1)	5.3 (1.1–24.6)*
≥ twice per day	2	154	1.00	1.00

*COR* Crude odds ratio, *AOR* Adjusted odds ratio

***Significant at *p*-value ≤0.001, ** Significant at *p*-value ≤0.01, * Significant at *p*-value ≤0.05

## Discussion

The overall prevalence of active trachoma was 11.8% (95% CI, 9.5–14.1). The factors associated with active trachoma were unprotected source of water; water consumption, improper latrine utilization, and frequency of washing.

The current prevalence of active trachoma was lower than other studies done in the region, Gonjokolela district of west Gojjam [18], Gazegibela district of Wagehemera zone [19], in Anckober [20] and Baso Liben district of east Gojjam [21]. Moreover, the overall prevalence of active trachoma was lower than studies done outside of the region in Zala district, Gamo Gofa zone [22] and Kersa district of Oromia zone [23] and in Ethiopia [24].

This study finding was also lower in prevalence than other studies done in Africa countries, Ethiopia, Guina, Uganda, Chad, Tanzania, Nigeria and Sudan [6]. This prevalence was also lower than the WHO threshold level used to determine trachoma as a sever public health problem according to WHO the threshold prevalence for TF/TI among children age 1–10 years old is 20% [8]. Several factors might have contributed to the observed reduction of the prevalence of active trachoma in the study district. These might be due to variations on annual continuous trachoma specific interventions such Azithromax distribution and health education, the implementation of Health Extension Packages particularly for increasing latrine coverage and open defecation free kebeles, and improved water supply coverage.

The WHO recommends a reduction of TF cases to less than 5% among children aged 1–9 to eliminate blinding trachoma. However, the number of active trachoma cases in the study districts was11.8%. This finding calls for an urgent need for mass distribution campaigns of antibiotics to reduce the transmission of trachoma in this district.

The study showed that those households who obtained water from unprotected source was 4.7 times more likely to develop active trachoma than those obtained water from protected source. This result was supported by a similar study done in Gazegibela district of Wagehemra Zone [19] and in Ankober, Ethiopia [20].

Water consumption per household was significantly associated with active trachoma. Those households which consume water from 40 to 60 l per household were 2.8 times more likely to develop active trachoma than those households that consume water more. This finding was similar with a study finding in Baso Liben District of East Gojjam, Ethiopia [21] and Areka Town, South Ethiopia [25].

The study also showed that improper latrine utilization was 3.2 times more likely to develop active trachoma than those used latrine properly. This is in agreement with studies done in Gonji Kolella district [18], North and South Wollo Zones, Ethiopia [26] Ancober, Ethiopia [20] and the study done by WHO [27]. This could be due to the presence of open-field feces as a breeding media for the trachoma fly vector Musca sorbens that leads to a higher chance of transmission.

This finding of study also showed that active trachoma was significantly associated with face washing habit. Children who wash their face once per day were 5.3 times more likely to be developing active trachoma as compared to children who wash their face greater than or equal two times per day. This finding was similar with studies done in Zala district, Southern Ethiopia [22], Wereilu district, Ethiopia [27] and Uganda [8]. Moreover, this finding was consistent with the systematic review and meta-analysis done in Ethiopia [28]. This is due to the fact that children with unclean faces could be more likely to spread ocular secretions infected with C. trachomatis.

Active trachoma was one of the public health problems in the western world-high income countries. Evidences indicate that these countries eliminate trachoma by improving the living condition and basic sanitation and simply eliminated by hygienic measures in the study area [29].

## Conclusion

The overall prevalence of active trachoma in Metema district was lower than other studies conducted in the region and WHO threshold prevalence of 20% to declare trachoma as a severe public health problem. This might herald that trachoma transmission is decreasing in Metema district. However, it is away from the elimination of trachoma as a public health problem in a community. Unprotected source of water, lower household water consumption, improper latrine utilization, and frequency of face washing once per day were the factors associated with active trachoma. So, all community members and stakeholders in the study district should further strengthen to work on trachoma prevention by implementing the WHO SAFE strategy- surgery for trichiasis, antibiotics, facial cleanliness and environmental improvement.

## Data Availability

Data will be made available upon requesting the primary author.

## References

[1] WHO (2011). Prevention of blindness and visual impairment (Priority Eye Diseases): Trachoma.

[2] Mariotti SP, Pararajasegaram R, Resnikoff S (2003). Trachoma: Looking forward to global elimination of trachoma by 2020 (GET 2020). Am J Trop Med Hyg.

[3] Edwards T, Harding-Esch EM, Hailu G, Andreason A, Mabey DC, Todd J, Cumberland P (2008). Risk factors for active trachoma and chlamydia trachomatis infection in rural Ethiopia after mass treatment with azithromycin. Trop Med Int Health.

[4] Polack S, Brooker S, Kuper H, Mariotti S, Mabey D, Foster A (2005). Mapping the global distribution of trachoma. Bull World Health Organ.

[5] Last AR BS, Weiss HA, Harding-Esh EM, Cassano E, Nabicassa M, et al. Risk factors for active trachoma and ocular Chlamydia trachomatis infection in treatment-Naı¨ve trachoma-Hyperendemic communities of the Bijago’ s archipelago, Guinea-Bissau. PLoS Negl Trop Dis. 2014;8(6):1–11. 10.1371/journal.pntd.0002900 PMC4072588 24967629

[6] Smith JLFR, Hooper PJ, Polack S, Cromwell EA (2013). The geographical distribution and burden of trachoma in Africa. PLOS NeglectedTrop Dis.

[7] DA Ferede AT, Tariku A, Adane AA (2017). Prevalence and determinants of active trachoma among preschool-aged children in Dembia District, Northwest Ethiopia. Infect Dis Pov.

[8] Weekly Epidemiological Record 2013 (2013). Prevention of Blindness and Visual Impairment.

[9] Australia. CDN: Trachoma (2014). CDNA national guideline for public health management of trachoma.

[10] WHO (2015). Trachoma Clinical characteristics and morbidity. *Factsheet N0 382*.

[11] Hamilton HVY (2013). Sightsavers: Water Aid: Washing away blinding trachoma; Sightsavers and WaterAid.

[12] Alemayehu M, Koye DN, Tariku A, Yimam K. Prevalence of active trachoma and its associated factors among rural and urban children in Dera Woreda, Northwest Ethiopia: a comparative cross-sectional study. Biomed Res Int. 2015;2015:1–8. 10.1155/2015/570898 PMC4390108 25954753

[13] WHO (2006). Trachoma control : aguide for programme managers.

[14] West SK (2003). Blinding trachoma: prevention with the safe strategy. Am J Trop Med Hyg.

[15] Berhane Y, Worku A, Bejiga A, Adamu L, Alemayehu W, Bedri A, Haile Z, Ayalew A, Adamu Y, Gebre T. Prevalence of trachoma in Ethiopia. Ethiopian J Health Dev. 2007;21(3):211–5.

[16] Gedefaw M, Shiferaw A, Alamrew Z, Feleke A, Fentie T, Atnafu K (2013). Current state of active trachoma among elementary school students in the context of ambitious national growth plan: the case of Ethiopia. Health.

[17] West Sheila KMB, Harran M, Gaydos Charlotte A, Quinn Thomas C (2011). Number of years of annual mass treatment with azithromycin needed to control trachoma in hyper-endemic communities in Tanzania. J Infect Dis.

[18] Nigusie A, Berhe R, Gedefaw M. Prevalence and associated factors of active trachoma among childeren aged 1–9 years in rural communities of Gonji Kolella district, West Gojjam zone, North West Ethiopia. BMC Res Notes. 2015;8(641):1–9. 10.1186/s13104-015-1529-6 PMC4632356 26530131

[19] Anteneh ZA, Getu WY. Prevalence of active trachoma and associated risk factors among children in Gazegibela district of Wagehemra Zone, Amhara region, Ethiopia: community-based cross-sectional study. Trop Dis, Travel Med Vaccines. 2016;2(5):1–7. 10.1186/s40794-016-0022-0 PMC5530921 28883949

[20] Golovaty I, Jones L, Gelaye B, Tilahun M (2009). Access to water source, latrine facilities and other risk factors of active trachoma in Ankober Ethiopia. PLoS One.

[21] Ketem K, Tiruneh M, Woldeyohannes D, Muluye D. Active trachoma and associated risk factors among children in Baso Liben District of East Gojjam, Ethiopia. BMC Public Health. 2012;12(1105):1–7. 10.1186/1471-2458-12-1105 PMC3543160 23259854

[22] Mengistu K, Shegaze M, Woldemichael K, Gesesew H, Markos Y (2016). Prevalence and factors associated with trachoma among children aged 1–9 years in Zala district, Gamo Gofa Zone, Southern Ethiopia. Dovepress J.

[23] Meseret E, Kariuki MM, Ilako DR, Yeshigeta G. Rapid Tracoma assesement in Kersa district, southwest Ethiopia. Ethiop J Health Science. 2013;23(1):1–9. PMC3613809 23559832

[24] Berhane Y, Worku A, Bejiga L, Adamu W, Alemayehu A (2007). Prevalence of trachoma in Ethiopia. Ethiop J Health Dev.

[25] Alambo MM, Lake EA, Bitew Workie S, Wassie AY. Prevalence of Active Trachoma and Associated Factors in Areka Town, South Ethiopia, 2018. Interdiscip Perspect Infect Dis. 2020;2020:8635191. 10.1155/2020/8635191 PMC7585665 33123195

[26] Tadesse B, Worku A, Kumie A, Yimer SA. The burden of and risk factors for active trachoma in the North and South Wollo Zones of Amhara Region, Ethiopia: a crosssectional study. Infect Dis Poverty. 2017;6(143):1–12. 10.1186/s40249-017-0358-3 PMC5632837 28988540

[27] Lemma E. Prevalence and risk Factors of trachoma among children of woreillu Woreda, South Wollo administrative zone. Ethiop J Health Sci. 2001;11(1).

[28] Gebrie A, Alebel A, Zegeye A, Tesfaye B, Wagnew F. Prevalence and associated factors of active trachoma among children in Ethiopia: a systematic review and meta-analysis. BMC Infect Dis. 2019;19(1073):1–12. 10.1186/s12879-019-4686-8 PMC6925509 31864307

[29] Abdou A, Munoz BE, Nassirou B, Kadri B, Moussa F, Baare I, Riverson J, Opong E, West SK (2010). How much is not enough? a community randomized trial of a water and health education programme for trachoma and ocular C.trachomatis infection in Niger. Trop Med Int Health.

